# Impacts of COVID-19 crisis and some related factors on the mental health of 37150 Vietnamese students: a cross-sectional online study

**DOI:** 10.1186/s12889-023-15317-3

**Published:** 2023-03-07

**Authors:** Chau Bao Duong, Nhi Van Tran, An Hoang Nguyen, Thong Nhat Le, Bien Huy Ha, Chau Ngoc Phuc Do, Khon Huynh, Thong Minh Le, Thao Phuong Nguyen, Hoai Thi Thu Nguyen

**Affiliations:** 1 School of Biotechnology, International University, Ho Chi Minh City, Vietnam; 2 Vietnam National University, Ho Chi Minh City, Vietnam; 3 Research Center for Infectious Diseases, International University, Ho Chi Minh City, Vietnam; 4 School of Biomedical Engineering, International University, Ho Chi Minh City, Vietnam

**Keywords:** COVID-19, Mental health, Lockdown status, Stress level, University students

## Abstract

**Background:**

University students are vulnerable to changes due to COVID-19 pandemic. Although warning has been made about the impact of this crisis on students’ mental health, there are barely any sufficient study. This work investigated how the pandemic affected the mental health of students at the Vietnam National University of Ho Chi Minh City (VNU-HCMC) and efficiency of available mental health supportive methods.

**Methods:**

An online survey was conducted among students at Vietnam National University of Ho Chi Minh City (VNU-HCMC) from October 18, 2021, to October 25, 2021. Microsoft Excel 16.51 (Microsoft, USA) and R language, Epi packages 2.44 and 4.1.1 (rdrr.io) were used for data analysis.

**Results:**

Thirty-seven thousand one hundred fifty students participated in the survey, including 48.4% female and 51.6% male. Online learning pressure was mainly recorded (65.1%). Many students suffered from sleeping disorders (56.2%). Some reported being victims of abuse (5.9%). Female students expressed a significantly higher level of distress than males, particularly the feeling of ambiguity about the purpose of life (*p*-value < 0.0001, OR: 0.94, 95% CI: [0.95–0.98]). Third-year students suffered higher stress levels than others, especially in online learning (68.8%, *p*-value < 0.05). Mental health statuses among students of different lockdown status regions were not significantly different. Therefore, lockdown status did not affect the stress levels of students which suggested that poor mental health outcomes seemed to root in the suspension of everyday university life rather than the prohibition of going out.

**Conclusions:**

During COVID-19, students experienced lots of stress and mental problems. These findings underscore the importance of academic and innovative activities, bringing attention to the needs of interactive study and extra-curricular activities.

**Supplementary Information:**

The online version contains supplementary material available at 10.1186/s12889-023-15317-3.

## Background

The coronavirus disease 2019 (COVID-19) pandemic caused by severe acute respiratory syndrome coronavirus 2 (SARS-CoV-2) is a leading global concern. In Vietnam, as of October 28, 2021, there were confirmed 959195 cases and 21953 deaths, as reported by WHO [1]. As in many countries, Vietnamese government officials decided to suspend schools, universities, and other educational institutions to prevent the rapid spread of COVID-19, which affected more than 1.6 million university students [2].

This rapidly evolving global crisis has caused widespread outbreaks that intensively impacted adolescents mental health and well-being [3–7], thus raising concerns about the public mental health and psychological adjustment [8–12]. Previous studies have reported that 10 to 20% of adolescence suffering from stress, anxiety, and depression [13, 14] and might be more likely to suffer mental health consequences after a traumatic and stressful event like COVID-19 global crisis [15]. However, existing studies have been focusing more on deep and long-term psychosocial vulnerability of the COVID-19 pandemic among small samples of medical staff and medical students [5, 16–21]. Therefore, it is neccessary to study on the impacts of COVID-19 on non-medical students at large scale.

COVID-19 pandemic-related factors include living near or under the epidemic areas, having family members and friends infected with COVID-19, having been in close contact with an individual with suspected COVID-19 or infected materials, and frequent exposure to information about COVID-19 on social media have been proved to be significantly associated with an increased risk of COVID-19-related mental health problems [17–21]. The negative impacts of implementing social distancing on mental health have been recorded [22, 23]. However, the consequences of lockdown status were still controversial [24–26]. Hence, it is crucial to shed some light on the psychological toll of COVID-19 on mental conditions of university students under many circumstances.

In Vietnam, there is still a lack of sufficient research about the psychiatric effects of the COVID-19 epidemic on university students [27]. M ost studies had small sample sizes, assessing single symptoms, or dealing with limited factors associated with mental health. To develop practical intervention strategies for protecting young people mental health, large-scale epidemiological studies are required to understand better COVID-19 better- related mental pressures [28].

The aim of this study was to assess the impacts of the COVID-19 pandemic on mental health of VNU-HCMC students. It firstly focused on mental health pressures and epidemiological characteristics of VNU-HCMC students during the COVID-19 outbreak in Vietnam, then analyzed the factors associated with risk of mental health problems and finally assessed the efficiency of mental health supportive methods.

## Methods

An online cross-sectional survey took place from October 18, 2021, to October 25, 2021, using Google forms to collect data to investigate mental and physical health during COVID-19 among VNU-HCM students. Participants were college and university students from nine universities of VNU-HCMC. Students were invited to join the survey voluntarily via social media platforms. Each university’s office of student services received the form and posted on their Facebook fanpage. The inclusion criteria for participants were as follows: (1) VNU-HCMC students having a valid student’s ID; (2) sufficient to read and understand Vietnamese questionnaire; (3) submitted only one response using the same IP address. Each question had multiple choices and must be answered in term of “required response item” before a participant moved to the next question. If the form contains unanswered questions, participants could not submit a form to avoid missing data entry. The questionnaire was not designed for multiple entries; thus, students were encouraged to complete the survey once. By returning the completed survey, participating students agreed to this research privacy policy and permitted their answers to appear without personally identified information. Written informed consent was obtained from all subjects and/or their legal guardian(s). All study procedures were performed in accordance with the Declaration of Helsinki and following Sex and Gender Equity in Research—SAGER guidelines for studying involving sex and gender consideration. This study was approved by the Board of Directors of Viet Nam National University – Ho Chi Minh City. Participation was voluntary and anonymous, and ethical principles were maintained throughout the survey. The researchers assured that the background and objectives of the study were explained carefully, and participation was encouraged without any pressure.

The questionnaire was designed into six sections, including 34 multiple choices questions. After performing a literature review of similar studies, it was developed to assess various aspects of mental health, emotional support, and social life of students (Additional file 1). The division of recent living locations, which was categorized by the lockdown status (based on the release by Centers for Disease Control and Prevention (CDC) in Vietnam, the latest update on 03/10/2021), was described in the Supporting information (Additional file 1: Table S1).

A 4-point scale, whose responses consisted of “Disagree,” “No idea,” “Agree,” and “Strongly agree” was used. The number of answers “Strongly agree” and “Agree” was later combined as “Agree.” Responses were counted and categorized using Microsoft Excel, version 16.51 (Microsoft, USA). The data were collected and analyzed using Microsoft Excel, version 16.51 (Microsoft, USA), and R version 4.1.1 and used two-sided *p* values throughout, adjusted by FDR [29]; *p* values less than 0.05 and 95% CIs were considered as the significant difference or significant association. Fisher’s Exact Test (based on counts) was applied to calculate the significant association as well as the risk relative (RR) and odds ratio (OR) within males and females, using the R language, Epi packages, version 2.44. The χ2 test was first performed for the factors “Academic year” and “Lockdown status”, and Fisher’s Exact Test was applied as the post-hoc test. Confidence intervals for the difference in proportions were estimated using the method described by Newcombe, 1998 that was included in Epi packages [30]. Column charts based on the Fisher’s Exact Test result were created by ggplot2 package version 3.3.5 to show the different impacts.

## Results

The total number of students participating in the survey was 37150, including 17969 female (48.4%) and 19181 male students (51.6%). The number of first-year students participating in the survey was the largest, with 12540 students (33.8%) and only 238 students in the sixth year (0.6%). In addition, most participants came from locations mixing the application of Directive 15 and Directive 16 (28646, 77.1%) and gave a good evaluation of the quality of online lectures (29612, 79.7%). The responses showed that more than 50% of surveyed students received one vaccine injection and had no relationship with people infected with COVID-19. In addition, there were 6880/37150 participants (18.5%) who were underprivileged students affected by the COVID-19 pandemic, 1783 (4.8%) participants who come from poor or near-poor households in which the primary financial source faced difficulties due to COVID-19. Data also reported 105/37150 (0.28%) participants whose parent(s) unfortunately passed away due to the pandemic. General information on participants in the survey was summarized in Table 1.

**Table 1:** Characteristics of the study participants

Items	Number of Respondents (%)	Items	Number of Respondents (%)
Total	37150 (100.0)	**Participated university**	
**Sex**		Bach Khoa University	9646 (26.0)
Female	17969 (48.4)	University of Science	12152 (32.7)
Male	19181 (51.6)	University of Social Sciences and Humanities	1044 (2.8)
**Academic year**		International University	3229 (8.7)
First-year	12540 (33.8)	University of Information Technology	95 (0.3)
Second-year	8370 (22.5)	University of Economics and Law	5442 (14.6)
Third year	8161 (22.0)	An Giang University	5421 (14.6)
Fourth-year	6555 (17.6)	Vietnam National University in Ben Tre	64 (0.2)
Fifth-year	1286 (3.5)	School of Political and Administration Sciences	57 (0.2)
Sixth year	238 (0.6)	**Good evaluation on the quality of online lectures**	
**Lockdown status of current living location**		Agree	29612 (79.7)
New normal	2755 (7.4)	No idea	4891 (13.2)
New normal + directive 19	663 (1.8)	Disagree	2647 (7.1)
New normal + directive 15 + 19	5086 (13.7)	**Relationship with COVID-19 infected persons**	
Directive 15 + 16	28646 (77.1)	Self	930 (2.5)
**Vaccination history**		Friends	6143 (16.5)
Not yet	4096 (11.0)	Acquaintances	12281 (33.1)
One dose	19445 (52.3)	Member of family	2476 (6.7)
Two dose	13609 (36.6)	No one	20944 (56.4)
**Financial status**			**Number of respondents (%)**
Underprivileged students affected by the COVID-19 pandemic			6880 (18.5)
Students from poor or near-poor households; The primary financial source of the family faces difficulties due to COVID-19			1783 (4.8)
Students whose parent(s) unfortunately passed away due to COVID-19			105 (0.28)
Students who do not fall into any of the listed categories			28382 (76.4)

**Fig. 1:**
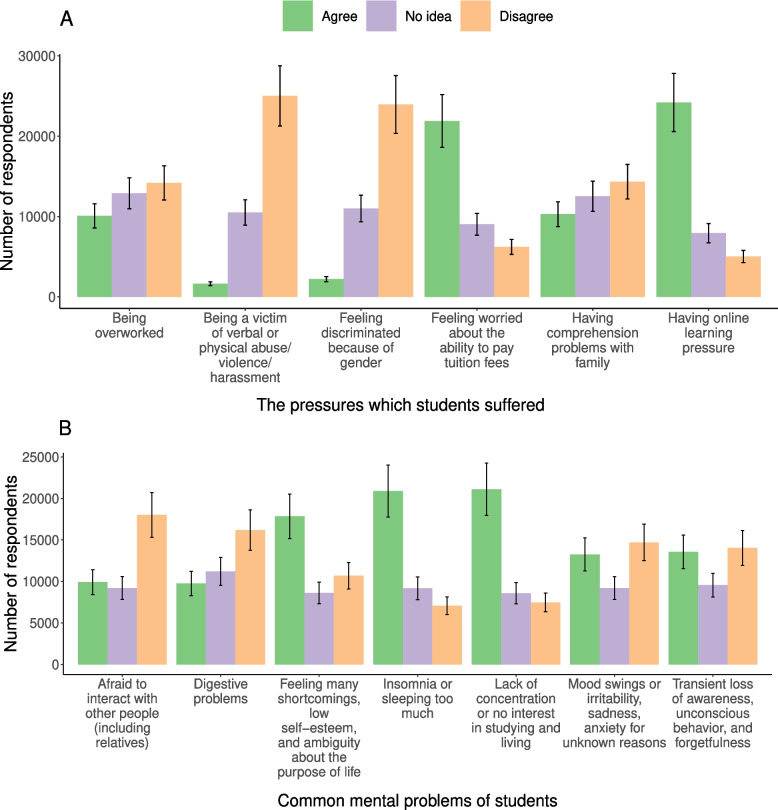
The pressures and common mental problems of students (%). **A** Common mental pressure. **B** Common mental problems

During the COVID-19 pandemic, there were 24195 (65.1%) students in the survey who reported suffering online learning pressure, followed by 21883 (58.9%) students who stressed about handling tuition fees. Notably, there were 1639 (4.4%) students who were victims of verbal or physical abuse/ violence/ harassment, and 2203 (5.9%) feeling discriminated against because of gender. (Fig. 1A, Additional file 2: Table S1).

Lack of concentration or interest in studying and living was quite common, with 21102 responses agreeing (56.8%). Patients with insomnia or excessive sleep were highly recorded (56.2%). 17845 participants (48.0%) confirmed shortcomings, low self-esteem, and ambiguity about the purpose of life during the COVID-19 pandemic (Fig. 1B, Additional file 2: Table S2).

**Fig. 2:**
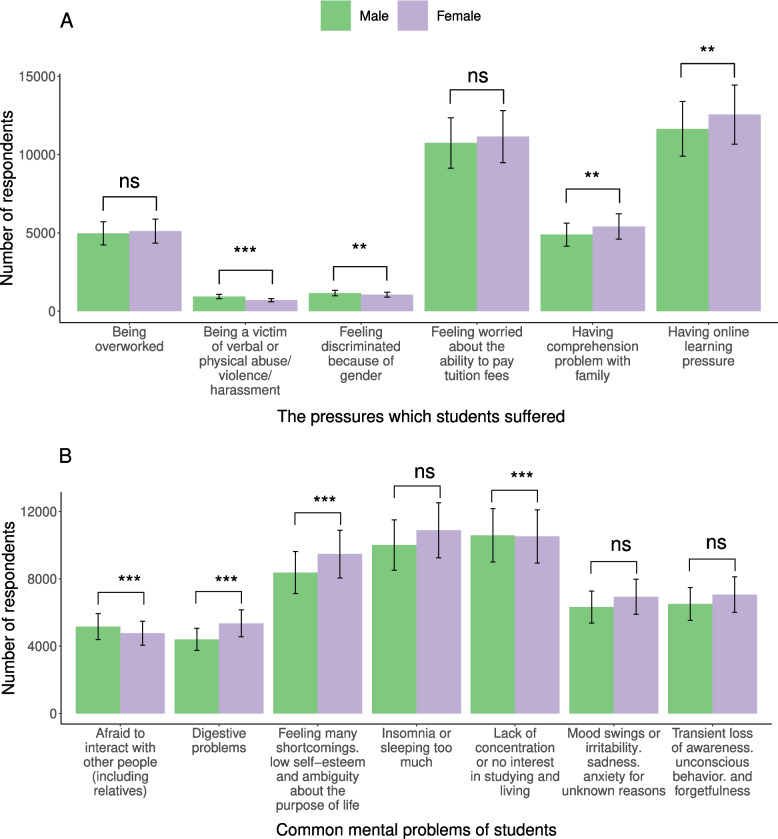
Differences in mental health pressures and common mental problems between male and female students. **A** Common mental health pressures. **B** Common mental problems. ***, **, * the level of significance (*p* ≤ 0.001—"***", 0.001 < *p* ≤ 0.01- "**", 0.01 < *p* ≤ 0.05—"*”, 0.05 < *p* ≤ 0.1—".", *p* > 0.1—ns); ns not significant

Results of Fisher’s Exact Test showed that female students were more stressed about online learning than male students (*p*-value = 0.008578, OR: 0.96, 95% CI: 0.93–0.99]) and had more comprehension problems with family (*p*-value = 0.006677, OR: 0.94, 95% CI: [0.9–0.98]). However, males were likely to be victim of verbal or physical abuse/ violence/ harassment than females (*p*-value = 1.60E-10, OR: 1.40, 95% CI: [1.27—1.54]). Furthermore, males also felt to be discriminated against because of gender more than females (*p*-value = 0. 002983, OR: 1.15, 95% CI: [1.06—1.26]) (Fig. 2A, Additional file 2: Table S4).

Concerning common mental problems, females were statistically more vulnerable to digestive problems (*p*-value = 2.16E-10, OR:0.87, 95% CI: [0.83–0.91]) than males. They more easily got feelings of many shortcomings, low self-esteem, and ambiguity about the purpose of life than males (*p*-value = 0.000111, OR: 0.94, 95% CI: [0.95–0.98]). The significantly higher risk for males was seen in the phenomenon of being afraid to interact with other people (including relatives) (*p*-value = 7.24E-14, OR: 1.18, 95% CI: [1.13–1.23]), lack of concentration, or no interest in studying and living (*p*-value = 3.77E-09, OR: 1.10, 95% CI: [1.06–1.13]) (Fig. 2B, Additional file 2: Table S6).

**Fig. 3:**
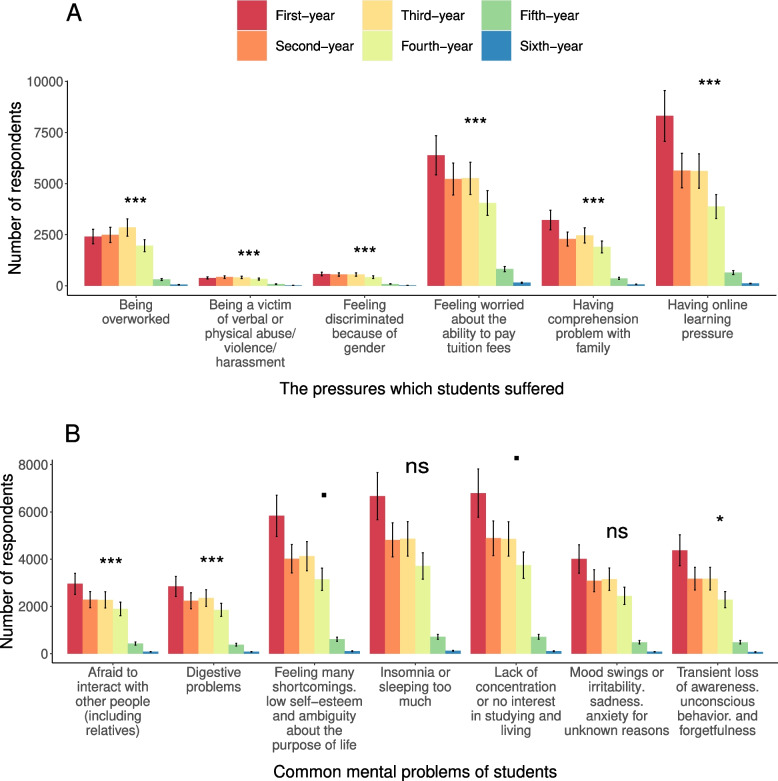
Differences in mental health pressures and common mental problems among students with different academic years. **A** Common mental health pressures. **B** Common mental problems. ***, **, *, the level of significance (p ≤ 0.001—"***", 0.001 < *p* ≤ 0.01- "**", 0.01 < *p* ≤ 0.05—"*”, 0.05 < *p* ≤ 0.1—".", *p* > 0.1—ns); ns not significant

Third-year students tended to be more stressed about online learning (*n* = 8161, 68.8%, *p*-value = 4.77E-47), highly conflicted with family in comprehension problems (30.2%, *p*-value < 0.001), overworked (34.9%, *p*-value = 1.00E-45) and nervous about the ability to pay the tuition fee (64.4%, *p*-value = 3.38E-06). Besides, it was notable that sixth-year students were more likely to suffer physical abuse/ violence/ harassment (*n* = 238, 7.6%, *p*-value = 1.368E-10) and discriminated against because of gender (7.1%, *p*-value < 0.001) (Additional file 2: Table S7, Fig. 3A). Besides, lack of concentration or having no interest in studying and living was significantly high in the third-year group (59.5%, *p*-value = 0.05). This group was also dominant in feeling many short-coming, low self-esteem, ambiguity about the purpose of life (50.6%, *p*-value = 0.02), transient loss of awareness, unconscious behavior, and forgetfulness (38.9%, *p*-value = 0.01). Digestive problems (*n* = 238, 33.6%, *p*-value = 1.52E-06) and avoiding interactions with others (34.5%, *p*-value = 1.94E-07) were frequently spread among sixth-year students (Additional file 2: Table S8, Fig. 3B). Fisher’s Exact Text was performed to confirm the discriminations between groups (Additional file 2: Table S9 and S10).

### Impact of lockdown status of current living locations on students' mental health

**Fig. 4:**
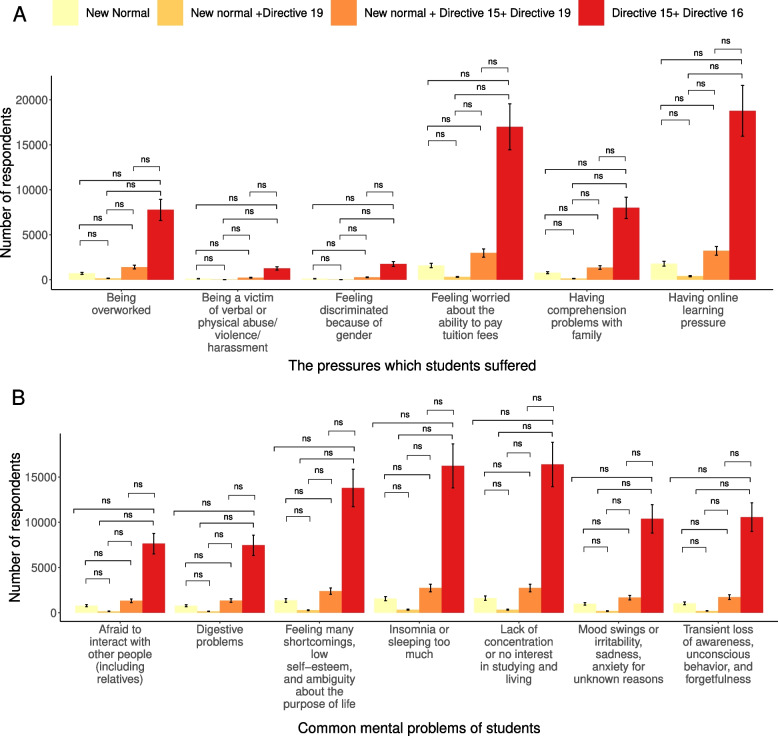
Differences in mental health pressures and common mental problems between students living under different lockdown conditions New normal Locations were ending the application of Directive 16 or Directive 15 or regional medical isolation; Directive 15,16, 19 Locations with Directive 19, 15, 16, respectively. **A** Common mental health pressures. **B** Common mental problems. ns Not significant

After performing the χ2 test, there was a disparity among students under different living locations based on the lockdown status (Table 2). They are different in having comprehension problems with family (*p*-value = 0.03), feeling discriminated against because of gender (*p*-value = 0.01), and feeling worried about the ability to pay tuition fees (*p*-value = 0.003). The difference was observed in those problems such as insomnia or sleeping too much (*p*-value = 0.045), lack of concentration, or no interest in studying and living (*p*-value = 0.006), mood swings or irritability, sadness, anxiety for unknown reasons (*p*-value < 0.001), transient loss of awareness, unconscious behavior, and forgetfulness (*p*-value = 0.001). However, there was no significant difference when the Fisher’s Exact Test was applied (Fig. 4).

**Table 2:** The impact of different lockdown statuses on common mental problems and mental pressures of students

		Number of "Agree" (%)				*p*-value
		New Normal	New normal + Directive 19	New normal + Directive 15 + Directive 19	Directive 15 + Directive 16	
Pressures	Having online learning pressure	1781 (64.6)	415 (62.6)	3220 (63.3)	18779 (65.6)	0.25
	Having comprehension problems with family	788 (28.6)	151 (22.8)	1358 (26.7)	7997 (27.9)	0.03
	Being a victim of verbal or physical abuse/ violence/ harassment	116 (4.2)	24 (3.6)	231 (4.5)	1268 (4.4)	0.705
	Feeling discriminated because of gender	127 (4.6)	29 (4.4)	276 (5.4)	1771 (6.2)	0.001
	Being overworked	721 (26.2)	160 (24.1)	1411 (27.7)	7782 (27.2)	0.278
	Feeling worried about the ability to pay tuition fees	1583 (57.5)	322 (48.6)	2977 (58.5)	17001 (59.3)	0.003
Problems	Insomnia or sleeping too much	1567 (56.9)	343 (51.7)	2747 (54)	16236 (56.7)	0.045
	Lack of concentration or no interest in studying and living	1617 (58.7)	346 (52.2)	2744 (54)	16395 (57.2)	0.006
	Afraid to interact with other people (including relatives)	790 (28.7)	169 (25.5)	1327 (26.1)	7638 (26.7)	0.164
	Mood swings or irritability, sadness, anxiety for unknown reasons	991 (36)	197 (29.7)	1684 (33.1)	10387 (36.3)	< 0.001
	Feeling many shortcomings, low self-esteem, and ambiguity about the purpose of life	1367 (49.6)	292 (44)	2394 (47.1)	13792 (48.1)	0.195
	Transient loss of awareness, unconscious behavior, and forgetfulness	1051 (38.1)	209 (31.5)	1729 (34)	10573 (36.9)	0.001
	Digestive problems	776 (28.2)	159 (24)	1358 (26.7)	7462 (26)	0.112

### Supportive methods and their impacts on students' mental health

Among the resources to support and maintain positive mental health during the COVID-19 pandemic, the majority of surveyed students agreed with home entertainment, such as reading books, newspapers, watching movies, and interacting on social networks (89.3%), while other respondents chose to stay connected with friends and family (84.5%). A remarkable number of students were concerned about improving physical health by working out at home (80.0%) and learning new skills or knowledge such as cooking or playing music (74.5%) (Table 3). Notably, there were reported that 9.8% of the respondents agreed to see a psychiatrist, illustrating a significant number of searches for specialized therapies for mental health and anxiety about COVID-19.

**Table 3:** Methods to maintain positive mental health during the COVID-19 pandemic

Methods	Number of respondents (%)		
	**Agree**	**No idea**	**Disagree**
Seeing a psychologist	3652 (9.8)	17593 (47.4)	15905 (42.8)
Working out at home	29736 (80.0)	5684 (15.3)	1730 (4.7)
Staying connected and chatting with friends and family	31384 (84.5)	4596 (12.4)	1170 (3.1)
Participating in local volunteering activities	12224 (32.9)	18150 (48.9)	6776 (18.2)
Learning new skills or knowledge (cooking, music, etc.)	27674 (74.5)	7366 (19.8)	2110 (5.7)
Entertainment at home: reading books, newspapers, watching movies, interacting on social networks	33191 (89.3)	3131 (8.4)	828 (2.2)

## Discussion

In Vietnam, poor mental health outcomes of university students due to the COVID-19 pandemic have not been previously investigated. This study is a large-scale, cross-sectional online survey to investigate the impacts of COVID-19 and associated factors on mental health of 37150 VNU-HCMC student during the pandemic in Vietnam. Our major results are summarized as fourth findings. First, the results of this survey showed that VNU-HCMC students mostly suffered online learning pressure and stressed about handling tuition fees. Second, the most common mental problems among VNU-HCMC students were sleeping disorders and lack of concentration and interest in life. Many students confirmed shortcomings, low self-esteem, and ambiguity about the purpose of life during the COVID-19 pandemic. Third, female students and third-year students were more prone to pressures and mental health problem during COVID-19 outbreak. Fourth, the level of lockdown status was not associated with negative mental health conditions of students.

Confirming similar findings, previous research on students' mental health worldwide during the COVID-19 pandemic further recorded that depression or anxiety is a risk factor for insomnia symptoms [5, 17]. The rate of VNU-HCMC students having insomnia was 56.2%, which was higher than that in a study about insomnia symptoms among adolescents and young adults in China during COVID-19 (23.2%) [17] and in the absence of this pandemic among Vietnamese students in Danang, Vietnam (31.1%) [31]. Our results revealed that university students were more likely to experience sleep disturbances and declines in sleep quality during COVID-19 due to previous poor sleep habits [31] and more hours free from school time. Lacking a consistent schedule due to school closure might accelerate mobile phone use, playing games and online shopping that can lead to sleep disorders. Besides, lack of concentration and interest in life, shortcomings, low self-esteem, and ambiguity about the purpose of life during the COVID-19 pandemic were also popular problems among VNU-HCMC students. Those problems might come from the pressures they had including online learning pressure and handling tuition fee, which were confirmed in this study. Among 37,150 participants, there was 18.5% (6880) underprivileged students affected by the COVID-19 pandemic, 4.8% (1783) students who their primary financial source of the family faced difficulties due to COVID-19. These circumstances might be one of reasons behind there was many students worried about tuition fees. Pressures might exacerbate the symptoms on insomnia, anxiety, and depression. Li and colleagues reported that under the strong stimulus of a stressor, individual with mental health problem would be threatened and express panic behavior [19]. University students, who lack the experience to cope and manage pressures, will be more likely to have depression, leading to an inevitable state of stress, causing emotional discomfort, pain, and mental fluctuations. Being homebound during the quarantine was reported to obscure students, leading to various psychiatric problems such as interpersonal sensitivity, hostility, and paranoia [32, 33]. Furthermore, students have a high risk of mental health disorders because of concerns about studying, graduating, and finding jobs [34, 35]. Notably, a small portion of students, who were victims of verbal or physical abuse/violence/ harassment, was also reported. Previous studies defined COVID-19 as a danger to family violence due to the sudden increase in poverty and family uncertainty, social isolation, limited social and healthcare services [36]. In addition, a small number of students reporting about the discrimination about gender during COVID-19 might belong to the LGBTQ + Community. A systematic preview revealed that these young people have been under difficult circumstances during COVD-19 crisis with unsupportive families due to sexual orientation and their previous disorders and traumas [37]. COVID-19 pandemic has significantly affected the quality of life of VNU-HCMC students, prolonged pressures and stressors can exacerbate anxiety and depression symptoms, so it is important to pay attention to students’ burdens and insomnia symptoms during and after COVID-19 outbreak. Public health center of local residence and university should provide psychosocial support and mental health services to their students, especially those who are at high risk of anxiety and depression.

In our study, we found two demographic factors that were associated with an increased risk of mental health problems among VNU-HCMC students. Even in the sample which had the majority of participants were male students, [34, 35] this survey revealed that female students were more vulnerable to mental health pressures during the COVID-19 pandemic. It strengthens the conclusion of previous research on the relationship between sex and and mental health, that females reported a higher prevalence of poor mental health outcomes before and during COVID-19 outbreak [4, 12, 38–41]. Female students might have sensitive stress responses to emergencies that can lead to an increased risk to experience pressures and stress [40–42]. This association might come from different physiological structures and functions between the male and female, as COVID-19 indirectly increased burden on women such as the family caring responsibilities while they had to work and study from home [24, 41]. The result showed that VNU-HCMC students in different academic years suffered mental pressures at different levels and t hird-year students were more likely to suffer mental health issues during COVID-19 pandemic [4, 42]. Third-year students, who was in the middle period of your academic career, were nervous about their future employment because they had to find an internship as well as to prepare for their upcoming thesis. While they had to finish specialized courses, adapt to new academic workloads, school expectations, and social relationships, the absence of interactive classes and shift to emergency online learning format would be the huge obstacles for them. Our finding was compatible with studies showed that seniors have higher rates of post-COVID-19 mental stress than first-year students [4, 24, 37, 40, 42]. So, female and third-year students need more support programs as well as more focused/targeted counseling services about planning their future career,  dealing with pressures, and building networking.

In this study, there was no significant discrimination between VNU-HCMC students under different level of restricted measures in Vietnam (Directive 15, 16, and Directive 19) and the “new normal” situation. This finding supports the conclusion that the strict level of lockdown status may not have a negative association with mental health problems [25, 26]. However, students living under the “new normal” situation reported the highest rate of almost listed pressures and problems (Table 2) except being a victim of verbal or physical abuse/ violence/ harassment, feeling discriminated because of gender, being overworked, and feeling worried about the ability to pay tuition fees. It might come from the reason that under the “new normal”, they can go out to avoid family violence or access different social health cares. Students living in the region without restricted measures might continue their part-time job, increase their ability to handle tuition fee. The concern was that, even under the "new normal," which has mild or no social distancing, students tended to suffer a significantly higher rate of stress and problems than the group which had undergone a strict lockdown. They still experienced a high prevalence of stress about learning online, difficulty comprehension with family, no interest, and uncertain feelings about life. The reasons behind the poor mental health outcomes of surveyed students might come from the prolonged disruption in daily activities and the absence of direct interactions and socialization, because even under the “new normal”, people still scared about the spreading of COVID-19. It might raise anxiety about the safety as they might feel they were not protected anymore and uncertainty about where probably the source of corona virus was. Because of the release of lockdown status, students under the “new normal” region might deal with increased distracting factors as their university still closed and they can go out anytime. There might be obstacles for them to manage and spend time for learning. So, students may face even more difficulties under the "new normal" in returning to their previous routine after a prolonged absence of a fixed schedule. The lack of interactive study and face-to-face communication would increase the incidence of insecurity, disorientation, and losing interest because of a long absence of extra-curricular activities. People should pay more attention for students under the “new normal” regions, introduce a strategy with safety guidelines for them to back a normal life free from COVID-19 traumas.

In this study, r esponses showed that social supports were mostly selected in maintaining the mental sustainability by university students during periods of COVID-19 crisis. It contributed to the findings that staying connected with social relationships has led to positive mental health status [23, 42]. Besides, students were more proactive in participating and creating positive things to contribute to a healthier mental lifestyle. Data during and after the pandemic has documented changes in young people's needs for psychiatric treatment and counseling by communicating with family and friends through Zoom and online calling. They have sought more professional solutions. According to the Organization for Economic Co-operation and Development (OECD), other mental health services have seen increase in usage in many countries, among which young users make up most calls to mental health hotlines [5].

Although this study provided a robust analysis with a large sample size (37150 respondents), the limited representativeness of this survey would lead to the over-representation of some participating universities where the survey was popular. There was no information to investigate the long-term mental health effects of the COVID-19 pandemic on students. Self-reported questionnaires were not enough to measure psychiatric symptoms at the clinical diagnosis level. The findings bring attention to health initiatives for students, which should include improvements in learning and living environments, such as developing resources to facilitate online guidance and lectures to offer strategies for managing anxiety.

## Conclusions

In conclusion, this study, which provided an analysis with a large sample size (37150 respondents), sufficiently investigated the self-perceived mental health of students at Vietnam National University as well as calculated the COVID-19 mental impact on Vietnam educational environment. While this study had some limitations the findings will contribute to health strategies which should include developing resources to facilitate online guidance and lectures to offer strategies for managing anxiety for students presently and in future.

## Supplementary Information

**Additional file 1.**

**Additional file 2.**

## Data Availability

The datasets used and/or analyzed during the current study are available from the corresponding author upon reasonable request.
