# Supplementary material for: Impacts of COVID-19 crisis and some related factors on the mental health of 37150 Vietnamese students: a cross-sectional online study
Source: BMC Public Health. 2023 Mar 7;23:445. doi: 10.1186/s12889-023-15317-3 (PMC9990976; doi:10.1186/s12889-023-15317-3)
Supplement: Supplementary file 1 — Additional file 1. [file 12889_2023_15317_MOESM1_ESM.docx]

**S1 – Additional details related to the study methods**

**Table S1. The details of current living locations based on the level of directive application [1-3].**

| **Group** | **Degree of strictness** | **General content** | **The directive content** |
| --- | --- | --- | --- |
| Directive 15 + 16 | Strict | Mixing between the application of Directive 16 and Directive 15. | Directive No. 15/CT-TTg (Directive 15): restricting movement from affected areas, canceling events with more than 20 people per room, banning gatherings of more than ten people outside offices, schools, and hospitals [1].  Directive No. 16/CT-TTg (Directive 16): temporarily suspending non-essential businesses and restaurants, banning public gatherings, and severely limiting transportation services from one area to another [2].  Directive No. 19/CT-TTg (Directive 19): loosening the restrictive measures that have been carried out to serve the prevention and control of pandemics in line with the pandemic developments and restore socio-economic activities based on ensuring reasonable control of the pandemic, especially in Hanoi City, Ho Chi Minh City, and big cities [3].  New normal: ending Directive 16 or Directive 15 or regional medical isolation. |
| New normal + Directive 15 + 19 | Conservative | Mixing between the "new normal" and the application of Directive 15. |  |
| New normal + Directive 19 | Moderate | Mixing between the "new normal" and the application of Directive 19/CT-TTg (Directive 19). |  |
| New normal | Liberal | No application of the directive. |  |

**References**

1. Differences between national government's Directives No 15, 16 and 19 on Covid-19 prevention and control 2021 [cited 2022 January 28]. Available from: <https://baodanang.vn/english/infographics/202009/differences-between-national-governments-directives-no-15-16-and-19-on-covid-19-prevention-and-control-3703977/>.

2. Directive 16 to be Applied in Southern Vietnamese Provinces as of July 19 2021 [cited 2022 January 15]. Available from: <https://vn.usembassy.gov/directive-16-to-be-applied-in-southern-vietnamese-provinces-as-of-july-19/>.

3. Directive 19/CT-TTg prevent, control the COVID-19 pandemic 2021 [cited 2022 January 15]. Available from: <https://english.luatvietnam.vn/directive-no-19-ct-ttg-on-continuing-to-implement-measures-to-prevent-and-control-the-covid-19-pandemic-in-182892-Doc1.html>.
