# Supplementary material for: Impacts of COVID-19 crisis and some related factors on the mental health of 37150 Vietnamese students: a cross-sectional online study
Source: BMC Public Health. 2023 Mar 7;23:445. doi: 10.1186/s12889-023-15317-3 (PMC9990976; doi:10.1186/s12889-023-15317-3)
Supplement: Supplementary file 2 — Additional file 2. [file 12889_2023_15317_MOESM2_ESM.docx]

**S2 – Additional Results**

Table of Contents

[1. The pressures and common mental problems of students 1](#_Toc115276414)

[2. Common mental pressures and mental problems between male and female students 2](#_Toc115276415)

[3. Impact of the academic year on student mental health. 3](#_Toc115276416)

# The pressures and common mental problems of students

**Table S1. The pressures which students suffered**

| **Pressures** | **Number of respondents (%)** | | |
| --- | --- | --- | --- |
|  | **Agree** | **No idea** | **Disagree** |
| Having online learning pressure | 24195 (65.1) | 7929 (21.3) | 5026 (13.5) |
| Having comprehension problems with family | 10294 (27.7) | 12514 (33.7) | 14342 (38.6) |
| Being a victim of verbal or physical abuse/ violence/ harassment | 1639 (4.4) | 10501 (28.3) | 25010 (67.3) |
| Feeling discriminated because of gender | 2203 (5.9) | 11004 (29.6) | 23943 (64.4) |
| Being overworked | 10074 (27.1) | 12894 (34.7) | 14182 (38.2) |
| Feeling worried about the ability to pay tuition fees | 21883 (58.9) | 9036 (24.3) | 6231 (16.8) |

**Table S2. Common mental problems of students**

| **Problems** | **Number of respondents (%)** | | |
| --- | --- | --- | --- |
|  | **Agree** | **No idea** | **Disagree** |
| Insomnia or sleeping too much | 20893 (56.2) | 9179 (24.7) | 7078 (19.1) |
| Lack of concentration or no interest in studying and living | 21102 (56.8) | 8578 (23.1) | 7470 (20.1) |
| Afraid to interact with other people (including relatives) | 9924 (26.7) | 9205 (24.8) | 18021 (48.5) |
| Mood swings or irritability, sadness, anxiety for unknown reasons | 13259 (35.7) | 9191 (24.7) | 14700 (39.6) |
| Feeling many shortcomings, low self-esteem, and ambiguity about the purpose of life | 17845 (48.0) | 8621 (23.2) | 10684 (28.8) |
| Transient loss of awareness, unconscious behavior, and forgetfulness | 13562 (36.5) | 9556 (25.7) | 14032 (37.8) |
| Digestive problems | 9755 (26.3) | 11207 (30.2) | 16188 (43.6) |

# Common mental pressures and mental problems between male and female students

**Table S3. Common mental pressures of male and female students**

| **Pressure** | **Number of “Agree” (%)** | |
| --- | --- | --- |
|  | **Male** | **Female** |
| Having online learning pressure | 11641 (60.7) | 12554 (69.9) |
| Feeling worried about the ability to pay tuition fees | 10739 (56.0) | 11144 (62.0) |
| Being overworked | 4969 (25.9) | 5105 (28.4) |
| Having comprehension problem with family | 4889 (25.5) | 5405 (30.1) |
| Feeling discriminated because of gender | 1152 (6.0) | 1051 (5.8) |
| Being a victim of verbal or physical abuse/ violence/ harassment | 934 (4.9) | 705 (3.9) |

**Table S4. The association of sex and common mental pressures on students**

| **Pressures** | **Group 1** | **Group 2** | **Adjusted p-value** | **95% CI for OR** | **OR** | **Significant p-value** |
| --- | --- | --- | --- | --- | --- | --- |
| Being a victim of verbal or physical abuse/ violence/ harassment | Male | Female | 1.60E-10 | 1.27-1.54 | 1.4 | *** |
| Feeling discriminated because of gender | Male | Female | 0.002983 | 1.06-1.26 | 1.15 | ** |
| Having online learning pressure | Male | Female | 0.008578 | 0.93-0.99 | 0.96 | ** |
| Having comprehension problem with family | Male | Female | 0.006677 | 0.9-0.98 | 0.94 | ** |
| Being overworked | Male | Female | 0.343754 | 0.98-1.07 | 1.02 | ns |
| Feeling worried about the ability to pay tuition fees | Male | Female | 0.392245 | 0.98-1.05 | 1.01 | ns |

**Table S5. Common mental problems of male and female students**

| **Problems** | **Number of “Agree” (%)** | |
| --- | --- | --- |
|  | **Male** | **Female** |
| Insomnia or sleeping too much | 10007 (52.5) | 10886 (60.6) |
| Lack of concentration or no interest in studying and living | 10582 (55.2) | 10520 (58.5) |
| Feeling many shortcomings, low self-esteem and ambiguity about the purpose of life | 8373 (43.7) | 9472 (52.7) |
| Transient loss of awareness. unconscious behavior. and forgetfulness | 6499 (33.9) | 7063 (39.3) |
| Mood swings or irritability. sadness. anxiety for unknown reasons | 6326 (30.0) | 6933 (38.6) |
| Afraid to interact with other people (including relatives) | 5159 (26.9) | 4765 (26.5) |
| Digestive problems | 4403 (23.0) | 5352 (29.8) |

**Table S6. The association between sex and common mental issues of students**

| **Problems** | **Group 1** | **Group 2** | **Adjusted p-value** | **95% CI for OR** | **OR** | **Significant p-value** |
| --- | --- | --- | --- | --- | --- | --- |
| Afraid to interact with other people (including relatives) | Male | Female | 7.24E-14 | 1.13-1.23 | 1.18 | *** |
| Lack of concentration or no interest in studying and living | Male | Female | 3.77E-09 | 1.06-1.13 | 1.1 | *** |
| Feeling many shortcomings, low self-esteem and ambiguity about the purpose of life | Male | Female | 0.000111 | 0.91-0.97 | 0.94 | *** |
| Digestive problems | Male | Female | 2.16E-10 | 0.83-0.91 | 0.87 | *** |
| Insomnia or sleeping too much | Male | Female | 0.242809 | 0.95-1.01 | 0.98 | ns |
| Mood swings or irritability. sadness. anxiety for unknown reasons | Male | Female | 0.221226 | 0.94-1.01 | 0.97 | ns |
| Transient loss of awareness. unconscious behavior. and forgetfulness | Male | Female | 0.362482 | 0.95-1.02 | 0.98 | ns |

# Impact of the academic year on student mental health.

**Table S7. Academic year and common mental pressures of students (χ2 test)**

| **Pressures** | **Number of “Agree” (%)** | | | | | | **p-value** |
| --- | --- | --- | --- | --- | --- | --- | --- |
|  | **First-year** | **Second-year** | **Third-year** | **Fourth-year** | **Fifth-year** | **Sixth-year** |  |
| Having online learning pressure | 8311 (66.3) | 5636 (67.3) | 5612 (68.8) | 3880 (59.2) | 645 (50.2) | 111 (46.6) | 4.77E-47 |
| Having comprehension problem with family | 3215 (25.6) | 2285 (27.3) | 2464 (30.2) | 1900 (29) | 361 (28.1) | 69 (29) | < 0.001 |
| Being a victim of verbal or physical abuse/ violence/ harassment | 382 (3) | 426 (5.1) | 407 (5) | 325 (5) | 81 (6.3) | 18 (7.6) | 1.368E-10 |
| Feeling discriminated because of gender | 576 (4.6) | 549 (6.6) | 552 (6.8) | 421 (6.4) | 88 (6.8) | 17 (7.1) | < 0.001 |
| Being overworked | 2408 (19.2) | 2494 (29.8) | 2849 (34.9) | 1964 (30) | 306 (23.8) | 53 (22.3) | 1.00E-45 |
| Feeling worried about the ability to pay tuition fees | 6385 (50.9) | 5222 (62.4) | 5258 (64.4) | 4051 (61.8) | 818 (63.6) | 149 (62.6) | 3.38E-06 |

**Table S8. Academic years and common mental problems of students, based on the result of the χ2 test**

| **Problems** | **Number of “Agree” (%)** | | | | | | **p- value** |
| --- | --- | --- | --- | --- | --- | --- | --- |
|  | **First-year** | **Second-year** | **Third-year** | **Fourth-year** | **Fifth-year** | **Sixth-year** |  |
| Insomnia or sleeping too much | 6665 (53.1) | 4816 (57.5) | 4861 (59.6) | 3716 (56.7) | 713 (55.4) | 122 (51.3) | 0.64 |
| Lack of concentration or no interest in studying and living | 6796 (54.2) | 4888 (58.4) | 4853 (59.5) | 3747 (57.2) | 710 (55.2) | 108 (45.4) | 0.05 |
| Afraid to interact with other people (including relatives) | 2957 (23.6) | 2285 (27.3) | 2278 (27.9) | 1893 (28.9) | 429 (33.4) | 82 (34.5) | 1.94E-07 |
| Mood swings or irritability. sadness. anxiety for unknown reasons | 4007 (32) | 3086 (36.9) | 3156 (38.7) | 2447 (37.3) | 481 (37.4) | 82 (34.5) | 0.07 |
| Feeling many shortcomings, low self-esteem and ambiguity about the purpose of life | 5834 (46.5) | 4015 (48) | 4129 (50.6) | 3150 (48.1) | 612 (47.6) | 105 (44.1) | 0.02 |
| Transient loss of awareness. unconscious behavior. and forgetfulness | 4375 (34.9) | 3175 (37.9) | 3174 (38.9) | 2286 (34.9) | 482 (37.5) | 70 (29.4) | 0.01 |
| Digestive problems | 2846 (22.7) | 2240 (26.8) | 2356 (28.9) | 1854 (28.3) | 379 (29.5) | 80 (33.6) | 1.52E-06 |

**Table S9. Academic year and common mental pressures of students (Fisher’s Exact Test).**

| Pressure | group1 | group2 | p.adj | CI_for.OR | OR | CI_for.RR | RR | sigP |
| --- | --- | --- | --- | --- | --- | --- | --- | --- |
| Being overworked | First-year_count | Second-year_count | 0.345628412 | 95% CI: 0.9-1.03 | 0.97 | 95% CI: 0.95-1.02 | 0.98 | ns |
|  |  | Third-year_count | 8.44E-07 | 95% CI: 0.79-0.9 | 0.85 | 95% CI: 0.88-0.95 | 0.92 | *** |
|  |  | Fourth-year_count | 5.27E-08 | 95% CI: 1.14-1.32 | 1.23 | 95% CI: 1.06-1.14 | 1.1 | *** |
|  |  | Fifth-year_count | 2.82E-284 | 95% CI: 6.91-8.96 | 7.87 | 95% CI: 1.72-1.83 | 1.77 | *** |
|  |  | Sixth-year_count | < 0.001 | 95% CI: 34.41-59.99 | 45.43 | 95% CI: 1.9-2.01 | 1.96 | *** |
|  | Second-year_count | Third-year_count | 9.16E-05 | 95% CI: 0.82-0.93 | 0.88 | 95% CI: 0.9-0.97 | 0.93 | *** |
|  |  | Fourth-year_count | 1.19E-10 | 95% CI: 1.18-1.36 | 1.27 | 95% CI: 1.08-1.16 | 1.12 | *** |
|  |  | Fifth-year_count | 1.05E-299 | 95% CI: 7.16-9.28 | 8.15 | 95% CI: 1.73-1.83 | 1.78 | *** |
|  |  | Sixth-year_count | < 0.001 | 95% CI: 35.65-62.11 | 47.06 | 95% CI: 1.9-2.01 | 1.96 | *** |
|  | Third-year_count | Fourth-year_count | 2.35E-25 | 95% CI: 1.35-1.56 | 1.45 | 95% CI: 1.15-1.22 | 1.18 | *** |
|  |  | Fifth-year_count | < 0.001 | 95% CI: 8.19-10.58 | 9.31 | 95% CI: 1.76-1.86 | 1.81 | *** |
|  |  | Sixth-year_count | < 0.001 | 95% CI: 40.77-70.88 | 53.75 | 95% CI: 1.91-2.02 | 1.96 | *** |
|  | Fourth-year_count | Fifth-year_count | 1.98E-206 | 95% CI: 5.61-7.34 | 6.42 | 95% CI: 1.67-1.79 | 1.73 | *** |
|  |  | Sixth-year_count | < 0.001 | 95% CI: 28.01-49.02 | 37.06 | 95% CI: 1.89-2.01 | 1.95 | *** |
|  | Fifth-year_count | Sixth-year_count | 3.28E-31 | 95% CI: 4.17-8 | 5.77 | 95% CI: 1.57-1.86 | 1.7 | *** |
| Being a victim of verbal or physical abuse/ violence/ harassment | First-year_count | Second-year_count | 0.232224301 | 95% CI: 0.76-1.06 | 0.9 | 95% CI: 0.87-1.03 | 0.95 | ns |
|  |  | Third-year_count | 0.520115473 | 95% CI: 0.79-1.11 | 0.94 | 95% CI: 0.89-1.06 | 0.97 | ns |
|  |  | Fourth-year_count | 0.089151216 | 95% CI: 0.98-1.41 | 1.18 | 95% CI: 0.99-1.18 | 1.08 | ns |
|  |  | Fifth-year_count | 2.28E-33 | 95% CI: 3.59-6.19 | 4.72 | 95% CI: 1.53-1.78 | 1.65 | *** |
|  |  | Sixth-year_count | 1.81E-65 | 95% CI: 12.97-34.73 | 21.22 | 95% CI: 1.78-2.05 | 1.91 | *** |
|  | Second-year_count | Third-year_count | 0.63169455 | 95% CI: 0.89-1.24 | 1.05 | 95% CI: 0.94-1.11 | 1.02 | ns |
|  |  | Fourth-year_count | 0.003141124 | 95% CI: 1.1-1.56 | 1.31 | 95% CI: 1.05-1.23 | 1.13 | ** |
|  |  | Fifth-year_count | 3.87E-40 | 95% CI: 4.02-6.87 | 5.26 | 95% CI: 1.56-1.81 | 1.68 | *** |
|  |  | Sixth-year_count | 1.46E-74 | 95% CI: 14.5-38.62 | 23.67 | 95% CI: 1.79-2.05 | 1.92 | *** |
|  | Third-year_count | Fourth-year_count | 0.016428617 | 95% CI: 1.05-1.5 | 1.25 | 95% CI: 1.02-1.21 | 1.11 | * |
|  |  | Fifth-year_count | 3.57E-37 | 95% CI: 3.84-6.58 | 5.02 | 95% CI: 1.55-1.8 | 1.67 | *** |
|  |  | Sixth-year_count | 1.87E-70 | 95% CI: 13.84-36.94 | 22.61 | 95% CI: 1.79-2.05 | 1.92 | *** |
|  | Fourth-year_count | Fifth-year_count | 6.05E-25 | 95% CI: 3.03-5.31 | 4.01 | 95% CI: 1.47-1.74 | 1.6 | *** |
|  |  | Sixth-year_count | 1.11E-53 | 95% CI: 10.98-29.7 | 18.06 | 95% CI: 1.75-2.05 | 1.9 | *** |
|  | Fifth-year_count | Sixth-year_count | 1.06E-07 | 95% CI: 2.52-8.05 | 4.5 | 95% CI: 1.38-1.93 | 1.64 | *** |
| Feeling discriminated because of gender | First-year_count | Second-year_count | 0.566215328 | 95% CI: 0.91-1.21 | 1.05 | 95% CI: 0.95-1.1 | 1.02 | ns |
|  |  | Third-year_count | 0.611563979 | 95% CI: 0.9-1.2 | 1.04 | 95% CI: 0.95-1.1 | 1.02 | ns |
|  |  | Fourth-year_count | 7.64E-05 | 95% CI: 1.17-1.59 | 1.37 | 95% CI: 1.08-1.24 | 1.16 | *** |
|  |  | Fifth-year_count | 3.06E-62 | 95% CI: 5.1-8.39 | 6.55 | 95% CI: 1.63-1.84 | 1.73 | *** |
|  |  | Sixth-year_count | 3.54E-107 | 95% CI: 20.64-55.62 | 33.88 | 95% CI: 1.83-2.06 | 1.94 | *** |
|  | Second-year_count | Third-year_count | 0.970557332 | 95% CI: 0.86-1.15 | 0.99 | 95% CI: 0.93-1.07 | 1 | ns |
|  |  | Fourth-year_count | 0.00095741 | 95% CI: 1.12-1.52 | 1.3 | 95% CI: 1.05-1.22 | 1.13 | *** |
|  |  | Fifth-year_count | 7.85E-58 | 95% CI: 4.86-8.01 | 6.24 | 95% CI: 1.62-1.84 | 1.72 | *** |
|  |  | Sixth-year_count | 1.51E-101 | 95% CI: 19.66-53.06 | 32.29 | 95% CI: 1.83-2.06 | 1.94 | *** |
|  | Third-year_count | Fourth-year_count | 0.000741776 | 95% CI: 1.12-1.53 | 1.31 | 95% CI: 1.06-1.22 | 1.13 | *** |
|  |  | Fifth-year_count | 2.56E-58 | 95% CI: 4.88-8.06 | 6.27 | 95% CI: 1.62-1.84 | 1.73 | *** |
|  |  | Sixth-year_count | 3.96E-102 | 95% CI: 19.77-53.34 | 32.47 | 95% CI: 1.83-2.06 | 1.94 | *** |
|  | Fourth-year_count | Fifth-year_count | 4.41E-37 | 95% CI: 3.69-6.21 | 4.78 | 95% CI: 1.54-1.78 | 1.65 | *** |
|  |  | Sixth-year_count | 2.87E-74 | 95% CI: 14.98-40.94 | 24.76 | 95% CI: 1.79-2.06 | 1.92 | *** |
|  | Fifth-year_count | Sixth-year_count | 3.27E-09 | 95% CI: 2.88-9.3 | 5.18 | 95% CI: 1.43-1.97 | 1.68 | *** |
| Feeling worried about the ability to pay tuition fees | First-year_count | Second-year_count | 1.65E-18 | 95% CI: 1.17-1.28 | 1.22 | 95% CI: 1.08-1.12 | 1.1 | *** |
|  |  | Third-year_count | 2.01E-17 | 95% CI: 1.16-1.27 | 1.21 | 95% CI: 1.07-1.12 | 1.1 | *** |
|  |  | Fourth-year_count | 3.98E-78 | 95% CI: 1.5-1.65 | 1.58 | 95% CI: 1.2-1.25 | 1.22 | *** |
|  |  | Fifth-year_count | < 0.001 | 95% CI: 7.21-8.45 | 7.81 | 95% CI: 1.74-1.81 | 1.77 | *** |
|  |  | Sixth-year_count | < 0.001 | 95% CI: 36.3-50.59 | 42.85 | 95% CI: 1.92-1.99 | 1.95 | *** |
|  | Second-year_count | Third-year_count | 0.800990072 | 95% CI: 0.95-1.04 | 0.99 | 95% CI: 0.97-1.02 | 1 | ns |
|  |  | Fourth-year_count | 3.76E-23 | 95% CI: 1.23-1.36 | 1.29 | 95% CI: 1.1-1.15 | 1.13 | *** |
|  |  | Fifth-year_count | < 0.001 | 95% CI: 5.88-6.93 | 6.38 | 95% CI: 1.69-1.76 | 1.73 | *** |
|  |  | Sixth-year_count | < 0.001 | 95% CI: 29.65-41.42 | 35.05 | 95% CI: 1.91-1.98 | 1.94 | *** |
|  | Third-year_count | Fourth-year_count | 2.06E-24 | 95% CI: 1.23-1.36 | 1.3 | 95% CI: 1.1-1.16 | 1.13 | *** |
|  |  | Fifth-year_count | < 0.001 | 95% CI: 5.92-6.98 | 6.43 | 95% CI: 1.7-1.77 | 1.73 | *** |
|  |  | Sixth-year_count | < 0.001 | 95% CI: 29.86-41.71 | 35.29 | 95% CI: 1.91-1.98 | 1.94 | *** |
|  | Fourth-year_count | Fifth-year_count | < 0.001 | 95% CI: 4.55-5.39 | 4.95 | 95% CI: 1.63-1.7 | 1.66 | *** |
|  |  | Sixth-year_count | < 0.001 | 95% CI: 22.96-32.19 | 27.19 | 95% CI: 1.89-1.97 | 1.93 | *** |
|  | Fifth-year_count | Sixth-year_count | 3.67E-78 | 95% CI: 4.51-6.68 | 5.49 | 95% CI: 1.61-1.78 | 1.69 | *** |
| Having comprehension problem with family | First-year_count | Second-year_count | 1.52E-24 | 95% CI: 1.32-1.5 | 1.41 | 95% CI: 1.14-1.2 | 1.17 | *** |
|  |  | Third-year_count | 5.49E-16 | 95% CI: 1.22-1.39 | 1.3 | 95% CI: 1.1-1.17 | 1.13 | *** |
|  |  | Fourth-year_count | 2.16E-51 | 95% CI: 1.58-1.81 | 1.69 | 95% CI: 1.22-1.29 | 1.26 | *** |
|  |  | Fifth-year_count | < 0.001 | 95% CI: 7.91-10.02 | 8.91 | 95% CI: 1.75-1.84 | 1.8 | *** |
|  |  | Sixth-year_count | < 0.001 | 95% CI: 36.53-59.43 | 46.59 | 95% CI: 1.91-2.01 | 1.96 | *** |
|  | Second-year_count | Third-year_count | 0.038734458 | 95% CI: 0.86-0.99 | 0.93 | 95% CI: 0.93-1 | 0.96 | * |
|  |  | Fourth-year_count | 1.52E-06 | 95% CI: 1.12-1.3 | 1.2 | 95% CI: 1.05-1.13 | 1.09 | *** |
|  |  | Fifth-year_count | 5.45E-238 | 95% CI: 5.59-7.16 | 6.33 | 95% CI: 1.67-1.78 | 1.73 | *** |
|  |  | Sixth-year_count | < 0.001 | 95% CI: 25.89-42.36 | 33.12 | 95% CI: 1.89-2 | 1.94 | *** |
|  | Third-year_count | Fourth-year_count | 3.97E-12 | 95% CI: 1.21-1.4 | 1.3 | 95% CI: 1.09-1.17 | 1.13 | *** |
|  |  | Fifth-year_count | 1.15E-268 | 95% CI: 6.04-7.71 | 6.83 | 95% CI: 1.69-1.8 | 1.74 | *** |
|  |  | Sixth-year_count | < 0.001 | 95% CI: 27.94-45.65 | 35.71 | 95% CI: 1.89-2 | 1.95 | *** |
|  | Fourth-year_count | Fifth-year_count | 1.26E-174 | 95% CI: 4.64-5.97 | 5.26 | 95% CI: 1.62-1.74 | 1.68 | *** |
|  |  | Sixth-year_count | < 0.001 | 95% CI: 21.48-35.29 | 27.54 | 95% CI: 1.87-1.99 | 1.93 | *** |
|  | Fifth-year_count | Sixth-year_count | 4.06E-34 | 95% CI: 3.91-6.99 | 5.23 | 95% CI: 1.55-1.82 | 1.68 | *** |
| Having online learning pressure | First-year_count | Second-year_count | 1.29E-76 | 95% CI: 1.42-1.54 | 1.47 | 95% CI: 1.17-1.21 | 1.19 | *** |
|  |  | Third-year_count | 4.25E-78 | 95% CI: 1.42-1.54 | 1.48 | 95% CI: 1.17-1.22 | 1.19 | *** |
|  |  | Fourth-year_count | 8.43E-243 | 95% CI: 2.05-2.24 | 2.14 | 95% CI: 1.34-1.39 | 1.36 | *** |
|  |  | Fifth-year_count | < 0.001 | 95% CI: 11.83-14.03 | 12.89 | 95% CI: 1.83-1.89 | 1.86 | *** |
|  |  | Sixth-year_count | < 0.001 | 95% CI: 61.94-90.51 | 74.87 | 95% CI: 1.94-2 | 1.97 | *** |
|  | Second-year_count | Third-year_count | 0.872169353 | 95% CI: 0.96-1.05 | 1 | 95% CI: 0.98-1.03 | 1 | ns |
|  |  | Fourth-year_count | 4.98E-49 | 95% CI: 1.38-1.53 | 1.45 | 95% CI: 1.16-1.21 | 1.18 | *** |
|  |  | Fifth-year_count | < 0.001 | 95% CI: 8-9.55 | 8.74 | 95% CI: 1.76-1.83 | 1.79 | *** |
|  |  | Sixth-year_count | < 0.001 | 95% CI: 41.93-61.48 | 50.77 | 95% CI: 1.93-2 | 1.96 | *** |
|  | Third-year_count | Fourth-year_count | 7.36E-48 | 95% CI: 1.38-1.52 | 1.45 | 95% CI: 1.16-1.21 | 1.18 | *** |
|  |  | Fifth-year_count | < 0.001 | 95% CI: 7.96-9.51 | 8.7 | 95% CI: 1.76-1.83 | 1.79 | *** |
|  |  | Sixth-year_count | < 0.001 | 95% CI: 41.75-61.22 | 50.56 | 95% CI: 1.92-2 | 1.96 | *** |
|  | Fourth-year_count | Fifth-year_count | < 0.001 | 95% CI: 5.48-6.6 | 6.02 | 95% CI: 1.67-1.76 | 1.71 | *** |
|  |  | Sixth-year_count | < 0.001 | 95% CI: 28.8-42.43 | 34.95 | 95% CI: 1.9-1.99 | 1.94 | *** |
|  | Fifth-year_count | Sixth-year_count | 1.43E-64 | 95% CI: 4.64-7.28 | 5.81 | 95% CI: 1.61-1.81 | 1.71 | *** |

**Table S10. Academic year and common mental problems of students (Fisher’s Exact Test).**

| factor | group1 | group2 | p.adj | CI_for.OR | OR | CI_for.RR | RR | sigP |
| --- | --- | --- | --- | --- | --- | --- | --- | --- |
| Afraid to interact with other people (including relatives) | First-year_count | Second-year_count | 3.74E-14 | 95% CI: 1.21-1.38 | 1.29 | 95% CI: 1.09-1.16 | 1.13 | *** |
|  |  | Third-year_count | 2.14E-14 | 95% CI: 1.21-1.39 | 1.3 | 95% CI: 1.1-1.16 | 1.13 | *** |
|  |  | Fourth-year_count | 6.68E-36 | 95% CI: 1.46-1.68 | 1.56 | 95% CI: 1.18-1.26 | 1.22 | *** |
|  |  | Fifth-year_count | < 0.001 | 95% CI: 6.16-7.71 | 6.89 | 95% CI: 1.7-1.79 | 1.75 | *** |
|  |  | Sixth-year_count | < 0.001 | 95% CI: 28.79-45.16 | 36.06 | 95% CI: 1.9-2 | 1.95 | *** |
|  | Second-year_count | Third-year_count | 0.95124615 | 95% CI: 0.93-1.08 | 1 | 95% CI: 0.97-1.04 | 1 | ns |
|  |  | Fourth-year_count | 8.17E-07 | 95% CI: 1.12-1.3 | 1.21 | 95% CI: 1.06-1.13 | 1.09 | *** |
|  |  | Fifth-year_count | 1.11E-211 | 95% CI: 4.74-5.98 | 5.33 | 95% CI: 1.63-1.74 | 1.68 | *** |
|  |  | Sixth-year_count | < 0.001 | 95% CI: 22.19-34.99 | 27.87 | 95% CI: 1.87-1.99 | 1.93 | *** |
|  | Third-year_count | Fourth-year_count | 1.27E-06 | 95% CI: 1.12-1.3 | 1.2 | 95% CI: 1.05-1.13 | 1.09 | *** |
|  |  | Fifth-year_count | 1.53E-210 | 95% CI: 4.73-5.96 | 5.31 | 95% CI: 1.63-1.74 | 1.68 | *** |
|  |  | Sixth-year_count | < 0.001 | 95% CI: 22.13-34.88 | 27.78 | 95% CI: 1.87-1.99 | 1.93 | *** |
|  | Fourth-year_count | Fifth-year_count | 6.56E-151 | 95% CI: 3.92-4.97 | 4.41 | 95% CI: 1.57-1.69 | 1.63 | *** |
|  |  | Sixth-year_count | < 0.001 | 95% CI: 18.35-29.05 | 23.09 | 95% CI: 1.86-1.98 | 1.92 | *** |
|  | Fifth-year_count | Sixth-year_count | 1.77E-40 | 95% CI: 4.01-6.83 | 5.23 | 95% CI: 1.56-1.8 | 1.68 | *** |
| Digestive problems | First-year_count | Second-year_count | 4.25E-12 | 95% CI: 1.19-1.36 | 1.27 | 95% CI: 1.08-1.15 | 1.12 | *** |
|  |  | Third-year_count | 3.35E-08 | 95% CI: 1.13-1.29 | 1.21 | 95% CI: 1.06-1.13 | 1.09 | *** |
|  |  | Fourth-year_count | 2.34E-32 | 95% CI: 1.43-1.65 | 1.54 | 95% CI: 1.17-1.25 | 1.21 | *** |
|  |  | Fifth-year_count | < 0.001 | 95% CI: 6.68-8.45 | 7.51 | 95% CI: 1.72-1.81 | 1.76 | *** |
|  |  | Sixth-year_count | < 0.001 | 95% CI: 28.32-44.69 | 35.58 | 95% CI: 1.89-2 | 1.95 | *** |
|  | Second-year_count | Third-year_count | 0.17417161 | 95% CI: 0.89-1.02 | 0.95 | 95% CI: 0.94-1.01 | 0.97 | ns |
|  |  | Fourth-year_count | 9.24E-07 | 95% CI: 1.12-1.3 | 1.21 | 95% CI: 1.06-1.13 | 1.09 | *** |
|  |  | Fifth-year_count | 2.90E-223 | 95% CI: 5.23-6.67 | 5.91 | 95% CI: 1.66-1.77 | 1.71 | *** |
|  |  | Sixth-year_count | < 0.001 | 95% CI: 22.24-35.25 | 28 | 95% CI: 1.87-1.99 | 1.93 | *** |
|  | Third-year_count | Fourth-year_count | 3.08E-10 | 95% CI: 1.18-1.37 | 1.27 | 95% CI: 1.08-1.16 | 1.12 | *** |
|  |  | Fifth-year_count | 1.02E-242 | 95% CI: 5.51-7.01 | 6.22 | 95% CI: 1.67-1.78 | 1.72 | *** |
|  |  | Sixth-year_count | < 0.001 | 95% CI: 23.4-37.06 | 29.45 | 95% CI: 1.88-1.99 | 1.93 | *** |
|  | Fourth-year_count | Fifth-year_count | 4.75E-161 | 95% CI: 4.32-5.54 | 4.89 | 95% CI: 1.6-1.72 | 1.66 | *** |
|  |  | Sixth-year_count | 2.08678506833967e-319 | 95% CI: 18.37-29.24 | 23.18 | 95% CI: 1.86-1.98 | 1.92 | *** |
|  | Fifth-year_count | Sixth-year_count | 2.41E-33 | 95% CI: 3.6-6.23 | 4.74 | 95% CI: 1.53-1.78 | 1.65 | *** |
| Feeling many shortcomings, low self-esteem and ambiguity about the purpose of life | First-year_count | Second-year_count | 8.11E-51 | 95% CI: 1.38-1.53 | 1.45 | 95% CI: 1.16-1.21 | 1.18 | *** |
|  |  | Third-year_count | 2.83E-44 | 95% CI: 1.35-1.48 | 1.41 | 95% CI: 1.15-1.2 | 1.17 | *** |
|  |  | Fourth-year_count | 1.52E-120 | 95% CI: 1.76-1.95 | 1.85 | 95% CI: 1.27-1.33 | 1.3 | *** |
|  |  | Fifth-year_count | < 0.001 | 95% CI: 8.71-10.43 | 9.53 | 95% CI: 1.78-1.84 | 1.81 | *** |
|  |  | Sixth-year_count | < 0.001 | 95% CI: 45.66-67.61 | 55.56 | 95% CI: 1.93-2 | 1.96 | *** |
|  | Second-year_count | Third-year_count | 0.32417614 | 95% CI: 0.92-1.03 | 0.97 | 95% CI: 0.96-1.01 | 0.99 | ns |
|  |  | Fourth-year_count | 8.65E-17 | 95% CI: 1.2-1.35 | 1.27 | 95% CI: 1.09-1.15 | 1.12 | *** |
|  |  | Fifth-year_count | < 0.001 | 95% CI: 5.97-7.21 | 6.56 | 95% CI: 1.7-1.78 | 1.74 | *** |
|  |  | Sixth-year_count | < 0.001 | 95% CI: 31.35-46.63 | 38.24 | 95% CI: 1.91-1.99 | 1.95 | *** |
|  | Third-year_count | Fourth-year_count | 7.60E-21 | 95% CI: 1.24-1.39 | 1.31 | 95% CI: 1.11-1.16 | 1.13 | *** |
|  |  | Fifth-year_count | < 0.001 | 95% CI: 6.14-7.41 | 6.75 | 95% CI: 1.7-1.78 | 1.74 | *** |
|  |  | Sixth-year_count | < 0.001 | 95% CI: 32.25-47.95 | 39.32 | 95% CI: 1.91-1.99 | 1.95 | *** |
|  | Fourth-year_count | Fifth-year_count | 1.07E-283 | 95% CI: 4.67-5.68 | 5.15 | 95% CI: 1.63-1.72 | 1.67 | *** |
|  |  | Sixth-year_count | < 0.001 | 95% CI: 24.55-36.66 | 30 | 95% CI: 1.89-1.98 | 1.94 | *** |
|  | Fifth-year_count | Sixth-year_count | 2.19E-61 | 95% CI: 4.62-7.35 | 5.83 | 95% CI: 1.61-1.81 | 1.71 | *** |
| Insomnia or sleeping too much | First-year_count | Second-year_count | 4.05E-45 | 95% CI: 1.32-1.45 | 1.38 | 95% CI: 1.14-1.18 | 1.16 | *** |
|  |  | Third-year_count | 7.24E-43 | 95% CI: 1.31-1.43 | 1.37 | 95% CI: 1.13-1.18 | 1.16 | *** |
|  |  | Fourth-year_count | 1.24E-125 | 95% CI: 1.71-1.88 | 1.79 | 95% CI: 1.26-1.31 | 1.28 | *** |
|  |  | Fifth-year_count | < 0.001 | 95% CI: 8.6-10.16 | 9.35 | 95% CI: 1.77-1.84 | 1.81 | *** |
|  |  | Sixth-year_count | < 0.001 | 95% CI: 45.53-65.55 | 54.63 | 95% CI: 1.93-2 | 1.96 | *** |
|  | Second-year_count | Third-year_count | 0.73917847 | 95% CI: 0.94-1.04 | 0.99 | 95% CI: 0.97-1.02 | 1 | ns |
|  |  | Fourth-year_count | 2.74E-22 | 95% CI: 1.23-1.37 | 1.3 | 95% CI: 1.1-1.16 | 1.13 | *** |
|  |  | Fifth-year_count | < 0.001 | 95% CI: 6.19-7.37 | 6.75 | 95% CI: 1.71-1.78 | 1.74 | *** |
|  |  | Sixth-year_count | < 0.001 | 95% CI: 32.84-47.45 | 39.48 | 95% CI: 1.91-1.99 | 1.95 | *** |
|  | Third-year_count | Fourth-year_count | 6.96E-24 | 95% CI: 1.24-1.38 | 1.31 | 95% CI: 1.11-1.16 | 1.13 | *** |
|  |  | Fifth-year_count | < 0.001 | 95% CI: 6.25-7.44 | 6.82 | 95% CI: 1.71-1.78 | 1.74 | *** |
|  |  | Sixth-year_count | < 0.001 | 95% CI: 33.15-47.89 | 39.84 | 95% CI: 1.91-1.99 | 1.95 | *** |
|  | Fourth-year_count | Fifth-year_count | < 0.001 | 95% CI: 4.76-5.7 | 5.21 | 95% CI: 1.64-1.72 | 1.68 | *** |
|  |  | Sixth-year_count | < 0.001 | 95% CI: 25.29-36.68 | 30.46 | 95% CI: 1.89-1.98 | 1.94 | *** |
|  | Fifth-year_count | Sixth-year_count | 1.35E-71 | 95% CI: 4.72-7.24 | 5.84 | 95% CI: 1.62-1.81 | 1.71 | *** |
| Lack of concentration or no interest in studying and living | First-year_count | Second-year_count | 3.52E-47 | 95% CI: 1.33-1.45 | 1.39 | 95% CI: 1.14-1.19 | 1.16 | *** |
|  |  | Third-year_count | 5.54E-49 | 95% CI: 1.34-1.46 | 1.4 | 95% CI: 1.14-1.19 | 1.17 | *** |
|  |  | Fourth-year_count | 3.20E-132 | 95% CI: 1.73-1.9 | 1.81 | 95% CI: 1.26-1.31 | 1.29 | *** |
|  |  | Fifth-year_count | < 0.001 | 95% CI: 8.8-10.41 | 9.57 | 95% CI: 1.78-1.84 | 1.81 | *** |
|  |  | Sixth-year_count | < 0.001 | 95% CI: 51.88-76.32 | 62.93 | 95% CI: 1.94-2 | 1.97 | *** |
|  | Second-year_count | Third-year_count | 0.79039206 | 95% CI: 0.96-1.06 | 1.01 | 95% CI: 0.98-1.03 | 1 | ns |
|  |  | Fourth-year_count | 1.28E-23 | 95% CI: 1.24-1.37 | 1.3 | 95% CI: 1.11-1.16 | 1.13 | *** |
|  |  | Fifth-year_count | < 0.001 | 95% CI: 6.31-7.51 | 6.88 | 95% CI: 1.71-1.78 | 1.75 | *** |
|  |  | Sixth-year_count | < 0.001 | 95% CI: 37.25-54.99 | 45.26 | 95% CI: 1.92-2 | 1.96 | *** |
|  | Third-year_count | Fourth-year_count | 2.41E-22 | 95% CI: 1.23-1.36 | 1.3 | 95% CI: 1.1-1.16 | 1.13 | *** |
|  |  | Fifth-year_count | < 0.001 | 95% CI: 6.27-7.46 | 6.84 | 95% CI: 1.71-1.78 | 1.74 | *** |
|  |  | Sixth-year_count | < 0.001 | 95% CI: 36.99-54.59 | 44.94 | 95% CI: 1.92-2 | 1.96 | *** |
|  | Fourth-year_count | Fifth-year_count | < 0.001 | 95% CI: 4.82-5.78 | 5.28 | 95% CI: 1.64-1.72 | 1.68 | *** |
|  |  | Sixth-year_count | < 0.001 | 95% CI: 28.51-42.23 | 34.69 | 95% CI: 1.9-1.99 | 1.94 | *** |
|  | Fifth-year_count | Sixth-year_count | 1.10E-76 | 95% CI: 5.25-8.23 | 6.57 | 95% CI: 1.64-1.83 | 1.74 | *** |
| Mood swings or irritability. sadness. anxiety for unknown reasons | First-year_count | Second-year_count | 4.70E-19 | 95% CI: 1.23-1.38 | 1.3 | 95% CI: 1.1-1.16 | 1.13 | *** |
|  |  | Third-year_count | 2.54E-16 | 95% CI: 1.2-1.34 | 1.27 | 95% CI: 1.09-1.15 | 1.12 | *** |
|  |  | Fourth-year_count | 3.79E-57 | 95% CI: 1.54-1.74 | 1.64 | 95% CI: 1.21-1.27 | 1.24 | *** |
|  |  | Fifth-year_count | < 0.001 | 95% CI: 7.51-9.24 | 8.33 | 95% CI: 1.74-1.83 | 1.79 | *** |
|  |  | Sixth-year_count | < 0.001 | 95% CI: 39.1-61.07 | 48.87 | 95% CI: 1.92-2 | 1.96 | *** |
|  | Second-year_count | Third-year_count | 0.49464062 | 95% CI: 0.92-1.04 | 0.98 | 95% CI: 0.96-1.02 | 0.99 | ns |
|  |  | Fourth-year_count | 2.51E-12 | 95% CI: 1.18-1.35 | 1.26 | 95% CI: 1.08-1.15 | 1.12 | *** |
|  |  | Fifth-year_count | < 0.001 | 95% CI: 5.77-7.14 | 6.42 | 95% CI: 1.68-1.78 | 1.73 | *** |
|  |  | Sixth-year_count | < 0.001 | 95% CI: 30.06-47.12 | 37.63 | 95% CI: 1.9-2 | 1.95 | *** |
|  | Third-year_count | Fourth-year_count | 1.24E-14 | 95% CI: 1.21-1.38 | 1.29 | 95% CI: 1.09-1.16 | 1.13 | *** |
|  |  | Fifth-year_count | < 0.001 | 95% CI: 5.9-7.3 | 6.56 | 95% CI: 1.69-1.78 | 1.74 | *** |
|  |  | Sixth-year_count | < 0.001 | 95% CI: 30.75-48.18 | 38.49 | 95% CI: 1.9-2 | 1.95 | *** |
|  | Fourth-year_count | Fifth-year_count | 1.05E-218 | 95% CI: 4.56-5.68 | 5.09 | 95% CI: 1.62-1.72 | 1.67 | *** |
|  |  | Sixth-year_count | < 0.001 | 95% CI: 23.79-37.44 | 29.84 | 95% CI: 1.88-1.99 | 1.94 | *** |
|  | Fifth-year_count | Sixth-year_count | 6.58E-49 | 95% CI: 4.52-7.62 | 5.87 | 95% CI: 1.6-1.83 | 1.71 | *** |
| Transient loss of awareness. unconscious behavior. and forgetfulness | First-year_count | Second-year_count | 1.77E-29 | 95% CI: 1.3-1.46 | 1.38 | 95% CI: 1.13-1.19 | 1.16 | *** |
|  |  | Third-year_count | 1.52E-29 | 95% CI: 1.3-1.46 | 1.38 | 95% CI: 1.13-1.19 | 1.16 | *** |
|  |  | Fourth-year_count | 5.88E-99 | 95% CI: 1.8-2.03 | 1.91 | 95% CI: 1.28-1.35 | 1.31 | *** |
|  |  | Fifth-year_count | < 0.001 | 95% CI: 8.2-10.05 | 9.08 | 95% CI: 1.76-1.84 | 1.8 | *** |
|  |  | Sixth-year_count | < 0.001 | 95% CI: 49.18-79.43 | 62.5 | 95% CI: 1.93-2.01 | 1.97 | *** |
|  | Second-year_count | Third-year_count | 1 | 95% CI: 0.94-1.06 | 1 | 95% CI: 0.97-1.03 | 1 | ns |
|  |  | Fourth-year_count | 8.98E-23 | 95% CI: 1.3-1.48 | 1.39 | 95% CI: 1.13-1.2 | 1.16 | *** |
|  |  | Fifth-year_count | < 0.001 | 95% CI: 5.92-7.33 | 6.59 | 95% CI: 1.69-1.78 | 1.74 | *** |
|  |  | Sixth-year_count | < 0.001 | 95% CI: 35.62-57.76 | 45.36 | 95% CI: 1.91-2.01 | 1.96 | *** |
|  | Third-year_count | Fourth-year_count | 1.03E-22 | 95% CI: 1.3-1.48 | 1.39 | 95% CI: 1.13-1.2 | 1.16 | *** |
|  |  | Fifth-year_count | < 0.001 | 95% CI: 5.92-7.32 | 6.59 | 95% CI: 1.69-1.78 | 1.74 | *** |
|  |  | Sixth-year_count | < 0.001 | 95% CI: 35.6-57.74 | 45.34 | 95% CI: 1.91-2.01 | 1.96 | *** |
|  | Fourth-year_count | Fifth-year_count | 2.49E-193 | 95% CI: 4.24-5.3 | 4.74 | 95% CI: 1.6-1.7 | 1.65 | *** |
|  |  | Sixth-year_count | < 0.001 | 95% CI: 25.57-41.71 | 32.66 | 95% CI: 1.88-2 | 1.94 | *** |
|  | Fifth-year_count | Sixth-year_count | 4.34E-54 | 95% CI: 5.22-9.08 | 6.89 | 95% CI: 1.63-1.87 | 1.75 | *** |
